# Supplementary figures and images for: The Risk of Gastrointestinal Bleeding between Non-Vitamin K Antagonist Oral Anticoagulants and Vitamin K Antagonists in the Asian Atrial Fibrillation Patients: A Meta-Analysis
Source: Int J Environ Res Public Health. 2020 Dec 27;18(1):137. doi: 10.3390/ijerph18010137 (PMC7795910; doi:10.3390/ijerph18010137)

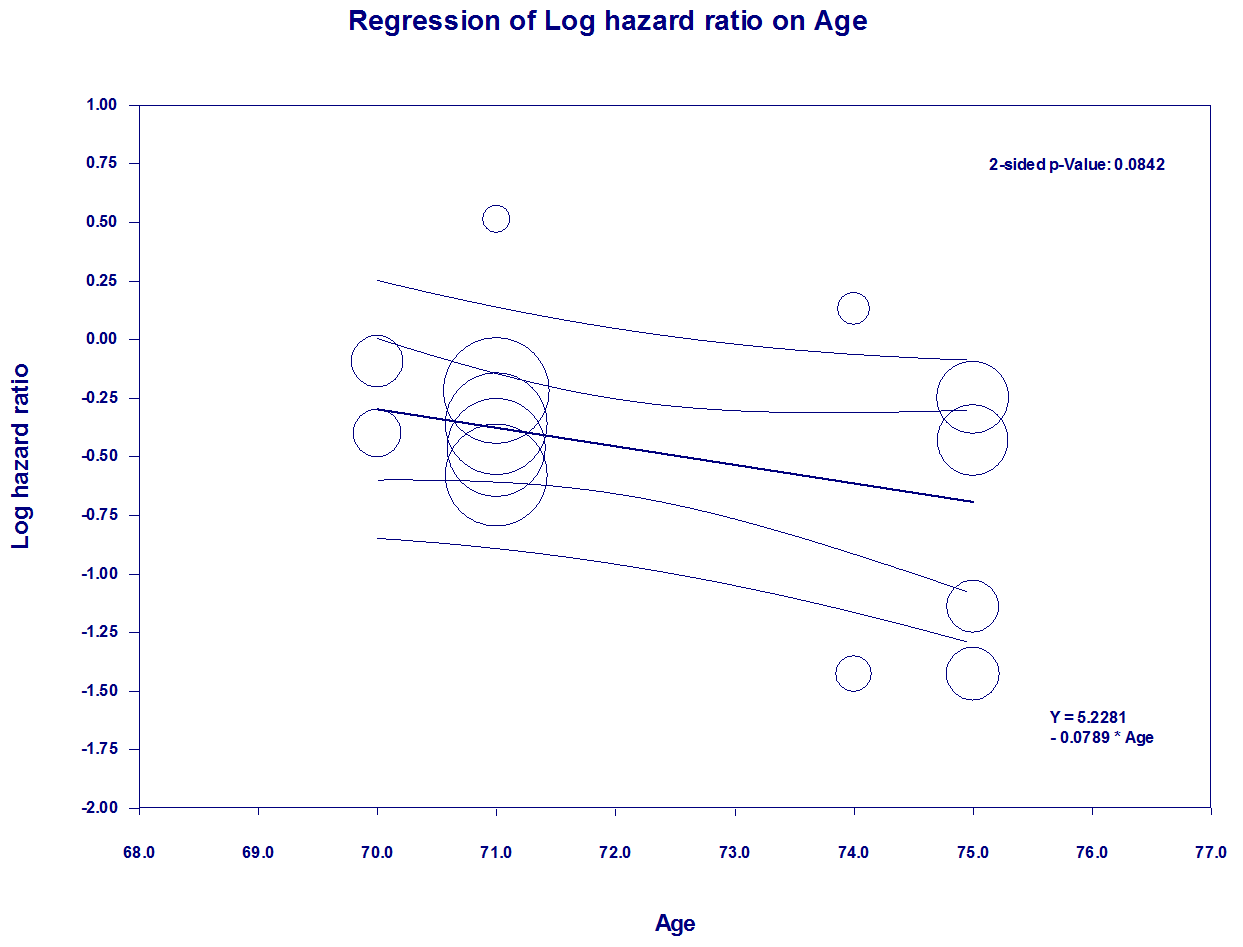

Supplement: Supplementary file 1 [file ijerph-18-00137-s001.zip › Figure S1.tif]

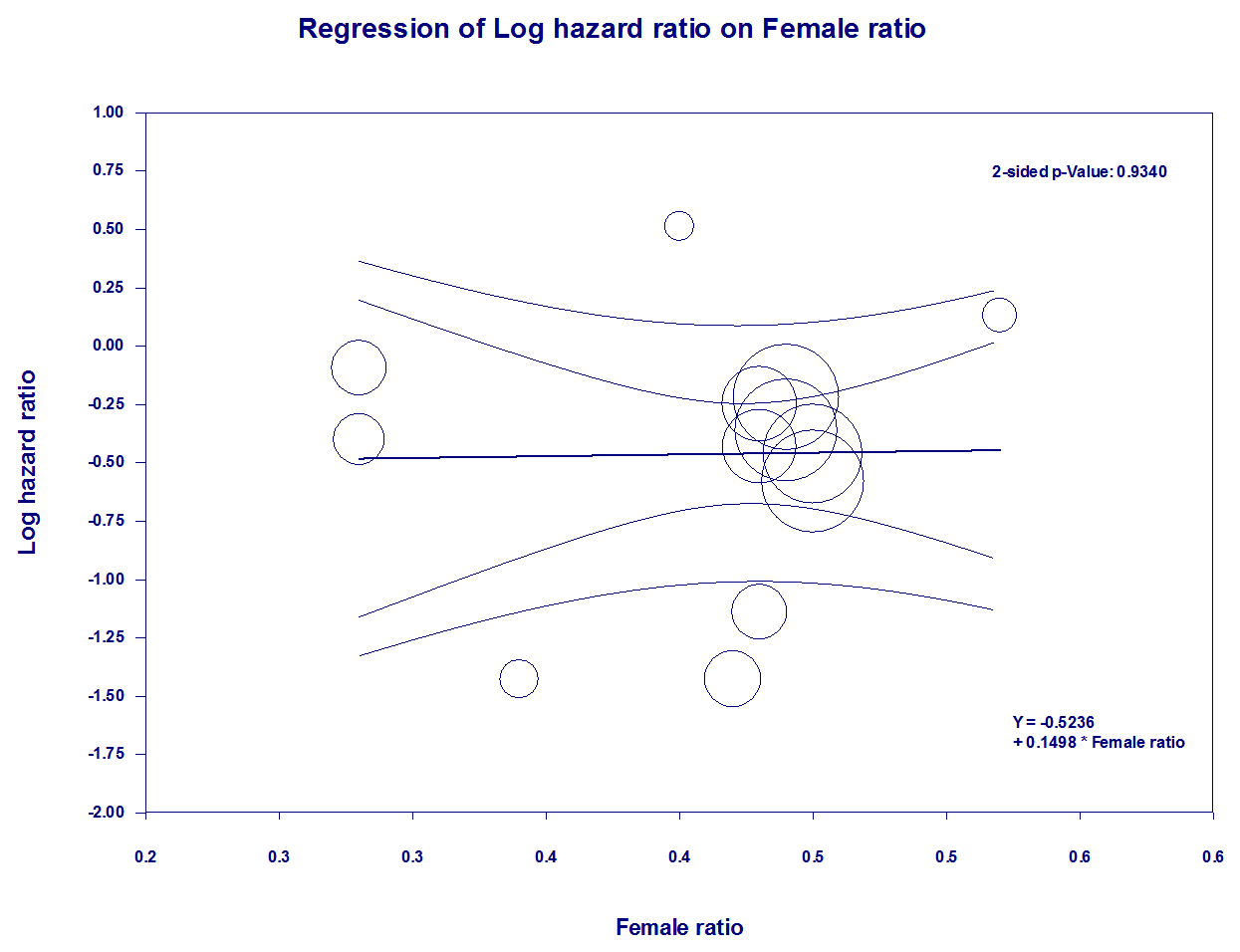

Supplement: Supplementary file 1 [file ijerph-18-00137-s001.zip › Figure S2.tif]

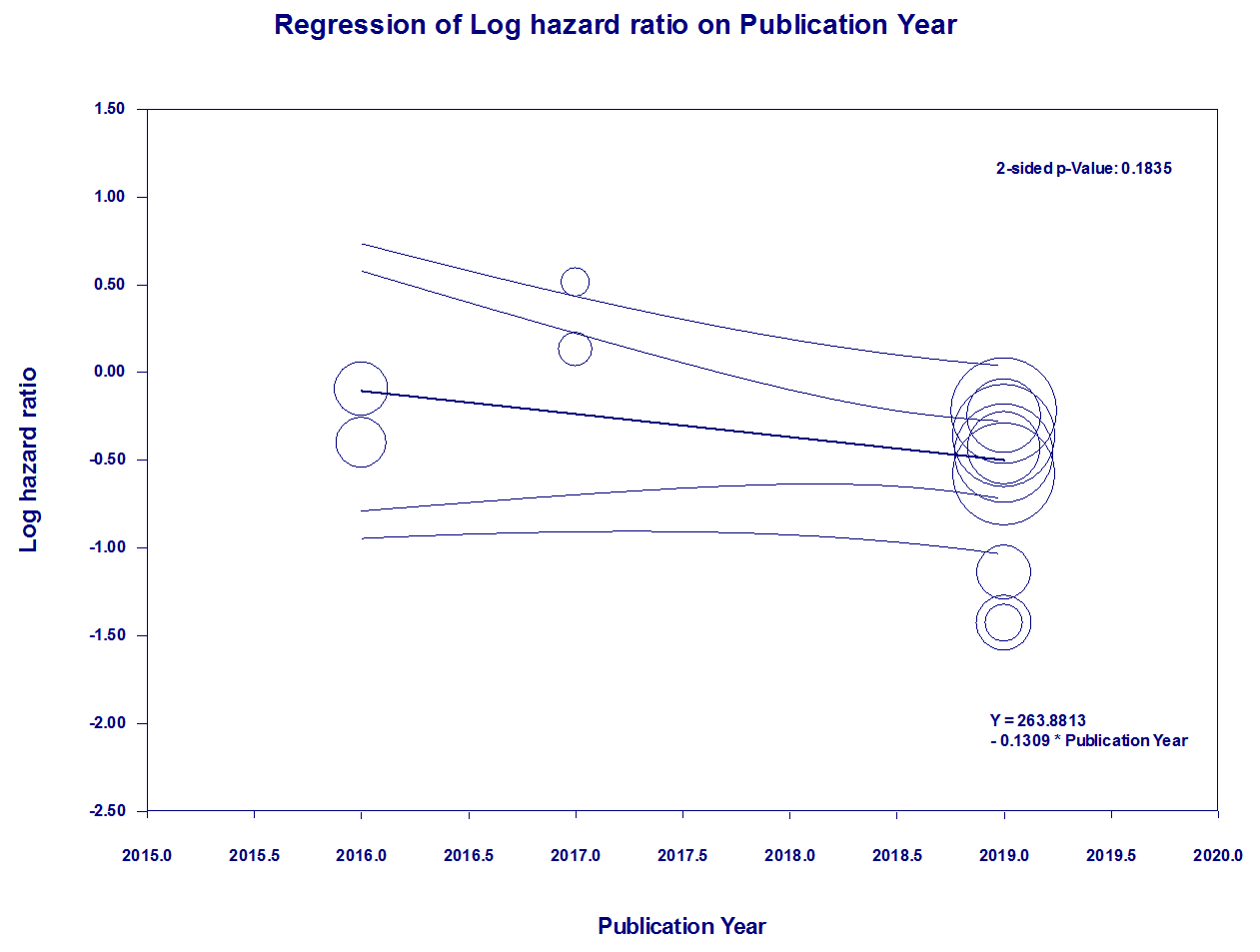

Supplement: Supplementary file 1 [file ijerph-18-00137-s001.zip › Figure S3.tif]

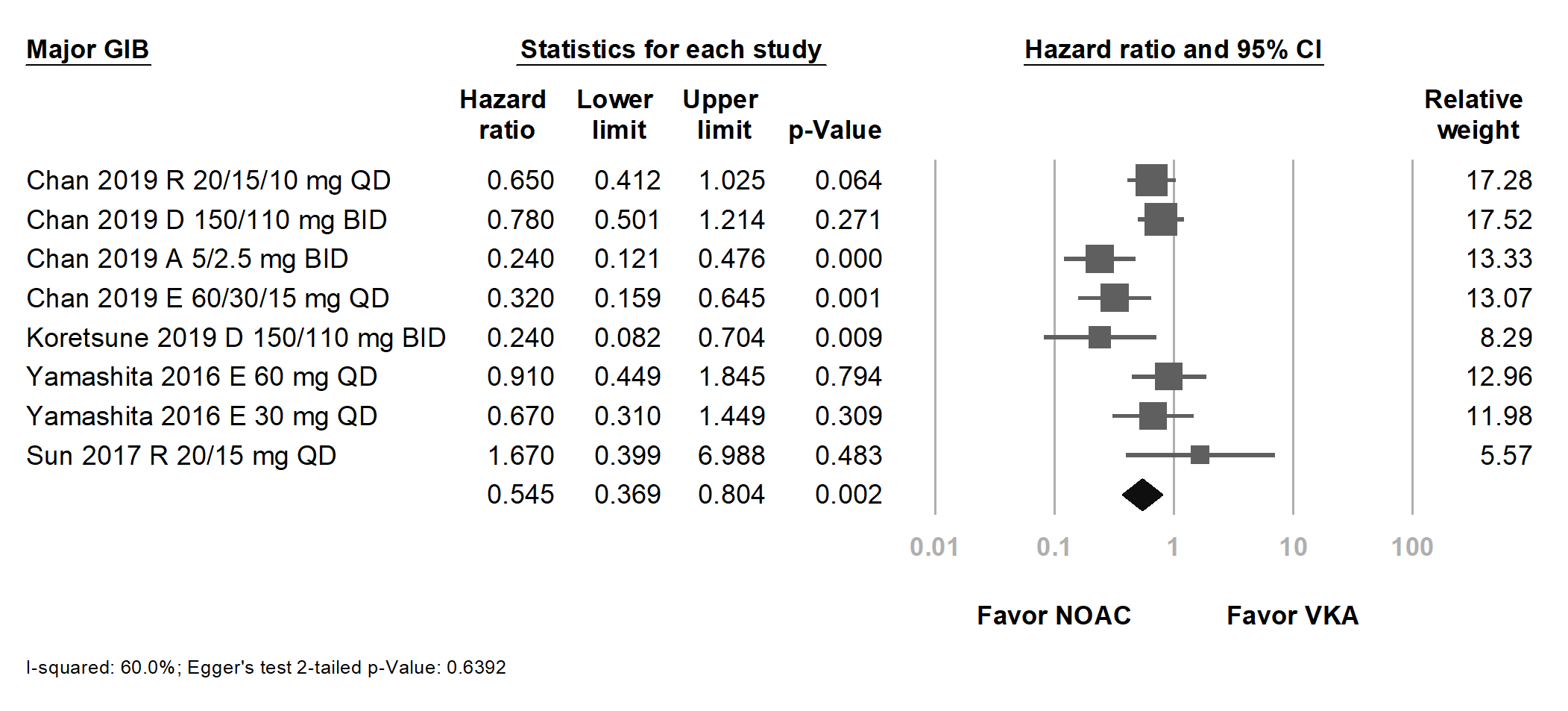

Supplement: Supplementary file 1 [file ijerph-18-00137-s001.zip › Figure S4.tif]

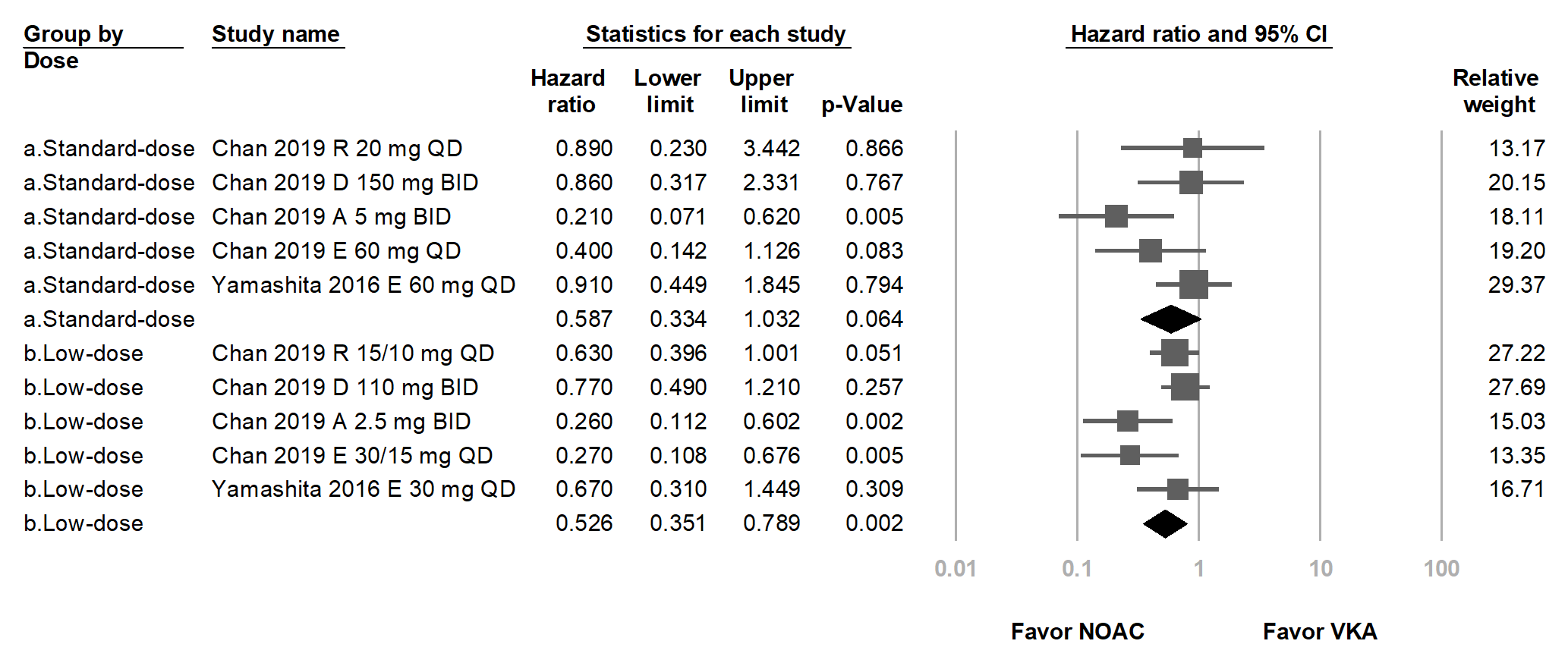

Supplement: Supplementary file 1 [file ijerph-18-00137-s001.zip › Figure S5.tif]
